# Face mask identification with enhanced cuckoo optimization and deep learning-based faster regional neural network

**DOI:** 10.1038/s41598-024-78746-z

**Published:** 2024-11-29

**Authors:** Binay Kumar Pandey, Digvijay Pandey, Mesfin Esayas Lelisho

**Affiliations:** 1 Department of Information Technology, College of Technology, Govind Ballabh Pant University of Agriculture and Technology Pantnagar, Uttrarakhand, India; 2 Department of Technical Education, Kanpur, Uttar Pradesh India; 3 Department of Statistics, College of Natural and Computational Science, Mizan-Tepi University, Tepi, Ethiopia

**Keywords:** *Faster R-CNN*, *IoT*, *UAV*, *OpenCV*, Infectious diseases, Data processing, Image processing

## Abstract

A mask identification and social distance monitoring system using Unmanned Aerial Vehicles (UAV) in the outdoors has been proposed for a health establishment. The above approach performed surveillance of the surrounding area using cameras installed in UAVs and internet of things technologies, and the captured images seem useful for tracking the entire environment. However, innate images from unmanned aerial vehicles show an adaptable visual effect in an uncontrolled environment, making face-mask detection and recognition harder. The UAV picture first had to be converted to grayscale, then its contrast was amplified. Image contrast was improved using Optimum Wavelet-Based Masking and the Enhanced Cuckoo Methodology (ECM). According to the contrast-enhanced image, Gabor-Transform (GT) and Stroke Width Transform (SWT) methods are used to derive attributes that help categorise mask-wearers and non-mask-wearers. Using the retrieved attributes, a Weighted Naive Bayes Classification (WNBC) detected masks in the images. Additionally, a deep neural network-based, the faster Region-Based Convolutional Neural Networks (R-CNN) algorithm combined with Adaptive Galactic Swarm Optimization (AGSO) is being used to identify appropriate and incorrect face mask wear in images, as well as to monitor social distancing among individuals in crowded areas. When the system recognises unmasked individuals, it sends their information to the doctor and the nearby police station. One unmanned aerial vehicle’s automated system alert people via speakers, ensuring social spacing. The problem involves a large percentage of appropriate and incorrect face mask wear using data from GitHub and Kaggle, including a training repository of 16,000 images and a testing data set of 12,751 images. To enhance the performance of the model’s learning, the methodology of 10-fold cross-validation will be used. Precision, recall, F1-score, and speed are then measured to determine the efficacy of the suggested approach.

## Introduction

The global COVID-19 pandemic has to be recognized as a serious threat. Considering the ongoing rise in occurrences, it is impractical for a governing body to keep track of them alone. The decrease in incidence of such an illness can only be possible with effective individual teamwork. The efficiency of minimizing transmission of viruses has been demonstrated extensively through the implementation of strategies such as distance from one another, hand sanitization, and the use of facial masks. Nonetheless, it is obvious that not all people follow these rules and regulations. Significant development is currently being made in the areas of Deep Learning (DL), Machine Intelligence (MI), Internet of Things (IoT)^1^, and Unmanned Aerial Vehicle (UAV). These developments possess the potential to accurately represent different global aspects, especially how populations are affected by physical barriers and the probability of individuals wearing face masks, among other things^2–5^. As a result, it is essential to develop an approach for dealing with issues that mask noncompliance and inattention to social distancing guidelines.

The IoT^6^technological advances sector has made substantial advancements, improving one’s capacity to carry out remote monitoring. The interconnectedness of computer-assisted elements across an internet^7^is an important aspect of the Internet of Things. To facilitate the transfer of data, each component will be allocated a unique role. Specifically, the method involves developing a face mask detection system using IoT technologies^8^. The proposed approach employs a dataset of images captured by an unmanned aerial vehicle (UAV) in congested regions to detect and distinguish between individuals wearing face masks or not, as well as measure the social distance between individuals. While the system recognizes a face without a mask, it promptly transmits details containing the individual’s image to each of the healthcare providers^9^and the police station nearest them. The process of identifying face masks in UAV camera images involves two tasks: detecting the mask and identifying whether it is appropriate or incorrect But prior to that, the captured UAV image must be beforehand processed. During initial processing, an image has become enlarged. The pre-processed visuals have been subsequently filtrated using a dynamic, unsharp masking approach. This approach employs an enhanced cuckoo search optimization methodology to select approximate coefficients. The application of an enhanced cuckoo search algorithm culminated in the design of an effective wavelet filter^10^, which is capable of constantly increasing contrast in images.

The image with higher contrast will be separated using the marker-controlled watershed segmentation method. The outcomes of segmentation are used to extract features via Gabor’s transform (GT). The features extracted are subsequently put into weighted Naive Bias Classification (WNBC) methodology to identify visuals with and without wearing face masks among the available UAV captured images. subsequently determining these two images, the one with the face mask is input into the faster RCNN, which combines with the OpenCV library. The faster R-CNN suggests using bounding boxes to determine the region of interest^11^ in order to distinguish between acceptable and erroneous facemask wear in UAV images. Furthermore, throughout the entire procedure of detecting appropriate and incorrect face mask wear in UAV images, whenever the data set comprises intricate, deformed images of individuals, the faster RCNN paired with the open OpenCV library operates less accurately. In order to prevent such circumstances, an AGSO is used along with faster RCNN to choose the most suitable weight parameter for faster RCNN. This enhances the overall accuracy of the entire procedure and emphasizes the novel component of the proposed methodology. In addition, the proposed approach is focused on monitoring social distancing among individuals using object recognition and traceability methods. the distance between the individuals is recognized in real time using bounding boxes, Raspberry Pi 4, OpenCV webcam, and UAV. The article is presented as follows: Sect. 2 explores the related work for the proposed approach. Section 3 describes the proposed methodology, while Sect. 4 examines the results and discussion. Finally, a conclusion has been defined in Sect. 5.

## Related work

Individuals have been devising numerous innovative ways to protect themselves from the COVID-19 disease. Scientists in fields such as the internet-of-things, machine learning^12^, computer-vision, blockchain, and others will be working on numerous methods to protect and cure people from the spread of this viral infection. In this paper, research was carried out using IoT^13^, a deep learning-based faster R-CNN method^14^, to identify face-masked individuals and also measure the social distancing among individuals. The whole section will explain related research in the area. As stated in^15^ scientific article, the objective is to make a face-mask, and also a social distancing detection framework as an entrenched imaging system. Throughout our sense, pre-trained models like MobileNet, ResNet Classifier, and VGG have been often chosen. Individuals who violated social detachment and were not donning face-mask have been nabbed. This article also includes a comparison of various face-mask identification and classification techniques.

To protect individuals from COVID-19^16^, employs a creative smart method based on the deep convolutional approach^17^. The suggested system will instantly discern whether or not individuals strictly adhere to safety procedures. A comparative evaluation of numerous deep learning^18^strategies to monitor illness from healthcare image analysis^19^has been completed throughout^20^. Further analysis^21^ could very well suggest an internet of things device for COVID-19 safeguards related to thermal identification, face-mask recognition, and social-distancing. The Arduino Uno is being used for infra-red sensing devices, whilst the Raspberry Pi has been used for mask-detection as well as social-distancing via vision-based methodologies.

^22^proposes a new face mask detection method based on image pre-processing, face-detection, and imaging super-resolution. When it comes to identifying balaclava wearers, the framework has proven to be highly precise. In^23^, a deep neural network-based framework for monitoring individuals and social distancing among individual people has been proffered, even in low-light conditions. In aspects of speed as well as accuracy, a methodology has been proven to be preferable to several previous methodologies. In^24^, A computer-vision-based methodology has been used to probe mask detection and social distancing in real-time. To supervise the various operations, the concept has been built on the Raspberry Pi. It has been discovered that it can save time but also decrease the propagation of COVID-19.

In^25^, for COVID-19 detection, an IoT-based deep learning has already been provided. The offered outline would have been used to identify pandemics besides utilising it for X-rays of the chest. This has been demonstrated to be exceedingly exact, providing healthcare workers with the timely identification of COVID-19. To identify the individuals’ wearing masks, describes a method predicated on Open-Source Computer Vision. The method has been proven to be effective, particularly in industrial uses. A thorough study of the predominant facets of COVID-19 has been proffered in^26^, and also the impacts of COVID-19 on Artificial Intelligence (AI), Unmanned Aerial Vehicles (UAVs), the Internet of Things, 5G, and other technologies will be addressed.

In^27^, the involvement of IoT as well as the relevant sensor systems for coronavirus monitoring and mitigation is discussed. This same research delves deeply into the e-health services premised on sensing devices for handling COVID-19, as well as successive networks again for the post-pandemic age^28^. discusses an evaluation of a technique that could be used to identify COVID-19. The review also describes the difficulties that will be encountered in incorporating such techniques in coming years. Deep learning associated with X-ray, In-Vitro Diagnostics (IVDs), and IoT-based devices were amongst the advancements considered towards following COVID-19 patients.

The paper^29^describes the use of deep learning based methodologies to solve few main problems like trying to detect people with or without masks, as well as predicting whether or not social distancing would be noticed as just an image segmentation issue. The exploratory results affirm HRNetV2’s ability to generate forecasts for assessing social distancing scores. A paper will be particularly useful when comparing the computational achievement of various edge inferencing methodologies, as well as the overall accuracy of such methodologies for such issue domains. The bench-marking methods used throughout the paper focus on secondary information and exploratory runs utilising established bench-marking techniques. Significant research will have been conducted in the fields of object recognition^30^ and conceptual segmentation using deep learning techniques.

## Proposed methodology

Face recognition is becoming increasingly important in content-based computer vision applications such as pattern classification^31^, monitoring, machine vision, fraud prevention, behavioural science, and neural nets^32^as a result of the global pandemic. During the investigation and competition, facial mask recognition likely garnered significant attention^33^. Mobile phone face-locking, signing with one’s face, and other applications have utilised face identification technology^34^. However, the identification of small and oblique face masks has been necessary in specific situations, such as areas with high crowd movement analysis, among others. Consequently, the pixel intensities in the image containing profiles and small faces are also incorporated. Convolutional neural networks (CNNs) are currently demonstrating enhanced performance in computer vision tasks, including object recognition, image labelling, and others^35^.

**Fig. 1:**
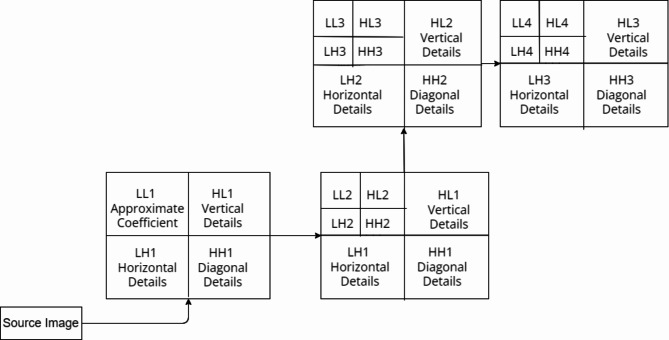
Level of decomposition.

**Fig. 2:**
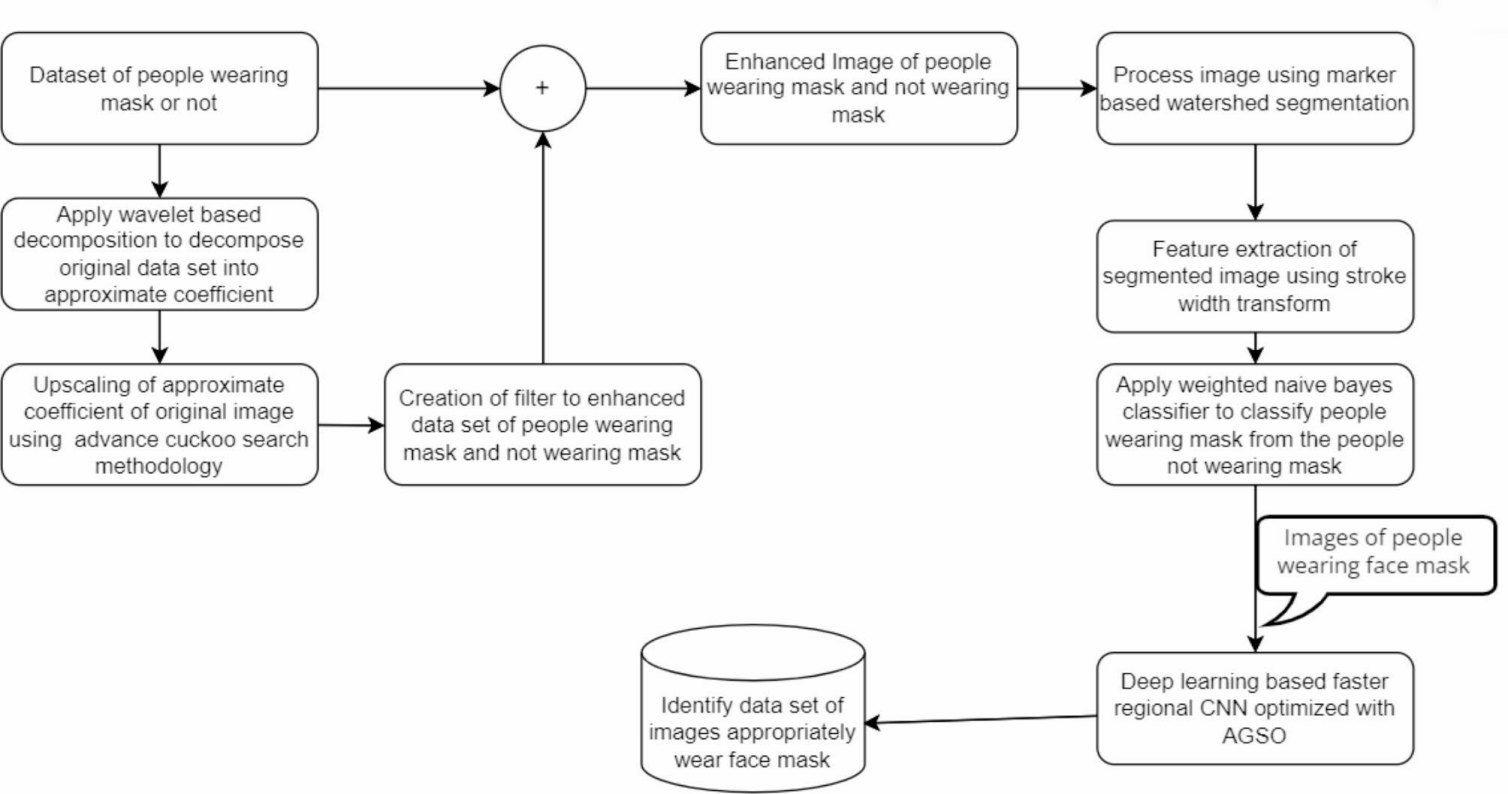
Flow diagram of the proposed methodology.

Consequently, scientists have employed these methodologies to implement several face recognition techniques^36,37^, such as R-CNN, fast R-CNN, and faster R-CNN. It enhances the generation of more timely and accurate discoveries. The proposed method illustrated in the Fig. 2, utilises the OpenCV^38^library along with the Raspberry Pi to develop an Internet of Things (IoT) system for identifying face masks. Additionally, it incorporates an UAV system for tracking social distancing^39^. A proposed system could effectively identify or verify an individual wearing mask or not by using a recorded video frame. The image^40^undergoes pre-processing, specifically employing a dynamic un-sharp masking technique. This technique involves selecting approximate coefficients using an optimisation methodology^41^. The Discrete Wavelet Transform (DWT) provided by Eq. 1 has been used to divide the original image into separate frequency bands that contain both accurate and approximate details. The levels of division appear to be categorised as Low-Level (LL), Low-High (LH), High-Low (HL), and High-High (HH). This encompasses three levels of comprehensive information, namely LH, HL, and HH, along with each level of approximation, LL. The low-level decomposition (LL) contains the highest amount of image data. The LH component contains the horizontal data, the HL component contains the vertical data, and the HH component contains the diagonal details. Following the initial stage of decomposition, the resulting elements are LL1, LH1, HL1, and HH1, respectively. LL1 has been selected for another round of decomposition in the exact same manner as depicted in the current image. The coefficient of approximation, LL2, will also be obtained in the next round. Similarly, an image is divided into up to four levels using the two-dimensional decomposition method. The obtained approximation coefficients, namely LL1, LL2, LL3, and LL4, as depicted in Fig. 1, are subsequently upscaled using advance cuckoo search algorithms and utilised in the construction of the filtration system to enhance the accuracy of the main image. The image would be decomposed into a maximum of 4 tiers due to the significant loss of detailed information after the 5th tier.

The coefficients approximated with the Inverse Discrete Wavelet Transformation (IDWT) as described in Eq. 2 have been calculated using Eq. 1.

1 $$\:W\phi\:(Jo, m, n)=\:\frac{1}{\sqrt{MN}}\sum\:_{x=0}^{M-1}\sum\:_{y=0}^{N-1}f\left(X,Y\right)\phi\:Jo,m,n\,(X, Y)$$

2 $$\:{f}_{(x,\:y)}^{A}=\:\frac{1}{\sqrt{MN}}{\sum\:}_{m}{\sum\:}_{n}w\varphi\:(Jo,m,n)\varphi\:Jo,m,n\,(X, Y)$$

The approximation coefficients are $\:W\varphi\:(Jo,m, n)$; J*₀* is the arbitrarily defined preliminary scale valuation; m, n, are the wavelet realm discrete variables. *f*_*(x, y)*_ is the temporal domains representation containing discrete variables as input *x*,* y* of size M*N, J₀=0,φ *J₀*,_*m, n*_*(*X, Y) is the scale-function, and $\:{f}_{(x,y)}^{A}$ are the recreated approximation coefficients. For the filter formation a wavelet approximate image has been used rather than original image.

The utilisation of an enhanced cuckoo search algorithm has resulted in the development of an efficient wavelet filter that is capable of dynamically enhancing image contrast. The image with improved contrast would be divided using the marker-controlled watershed technique. The segmentation results are used to extract features using Gabor’s Transform (GT). These extracted features are then inputted into the weighted Naive Bias Classification (WNBC) to determine the presence or absence of a facemask. The Region of Interest (ROI) is acknowledged if it is present. Subsequently, ROI pooling has been employed to collect and resize image features for the purpose of creating a novel feature extraction^42^. Consequently, it was determined to utilise classification^43^and regression on each region of the new feature map in order to predict component values for the bounding box once more. R-CNN exhibit a lower object detection capability^44^compared to faster RCNN. Moreover, alternative connectivity is employed to identify a specific area of recommendation in specific cases of faster R-CNN. Due to the time-consuming nature of R-CNN and its additional computing requirements^45^, a more advanced iteration called faster R-CNN was created. In order to compensate for aspect ratio variations, faster R-CNN employs OpenCV bounding boxes to figure out the region of interest in UAV images for recognizing proper and erroneously face mask wear.

Although OpenCV was originally developed to provide a common framework for machine learning methods^46^and improve the practical application of machine perception and recognition, the OpenCV library has demonstrated remarkable effectiveness in identifying and recognising proper and not proper wear of the masks. The library encompasses over 2500 optimisation techniques^47^, comprising both conventional and state-of-the-art deep learning as well as computer-vision methodologies. The OpenCV libraries have employed optimisation methodologies to perform various tasks such as face image recognition^48^, interpretation and classification of living organism movement in videos, artefact recognition, webcam motion tracking, 3D approach extraction, creation of point clouds using stereo camera systems, image stitching to produce a composite image, identification of related images^49^ from a database, removal of bloodshot eyes from images, and assessment of physical movements such as eye movements, palpation, and gait. However, if the data set comprises complex, perverted framed visuals of people, the faster RCNN teamed with the open OpenCV library approach utilized for recognizing appropriate and erroneously face-mask wear within UAV images appears to operate less accurately. In order to prevent these kinds of scenarios, an AGSO is utilized with a faster RCNN to figure out the optimal weight parameter to be used for the faster RCNN. AGSO’s parameters have been continually adjusting employing a fuzzier technique. This AGSO method has been motivated by the shifting motion of stars and galaxies under the impact of the force of gravity. The AGSO works in two phases: exploration and extraction. Throughout the exploration stage, the subpopulation of particles searches within vector space for a best-case scenario. during the exploitation stage, the most appropriate solution for every subpopulation moves closer to the globally most effective. Also, such galaxies are treated as points of mass on an adequate scale. Once again, these achieved galaxies come together in large numbers in order to get the galaxies extremely grouped. As a result, the overall accuracy of identifying proper and erroneous face mask wear in UAV images improves.

In this work, it was found that a UAV primarily used for aerial imagery has the potential to replace certain parts in order to facilitate amphibious operations. Additionally, a Raspberry Pi kit can be used for real-time picture identification. It has potential applications in real-world scenarios such as data collection studies, aerial photography, safety regulations, and tracking^50^community distances in crowded areas^51^. The UAV could effortlessly discern the spatial arrangement among individuals and objects, including various entities such as swans, animals, plants, vegetation, and houses. This serves as an intimidation to government officials and individuals in positions of authority.

Unmanned Aerial Vehicles (UAVs), particularly when combined with the integration of the “Internet of Things” (IoT), are regarded as dependable aerial platforms for efficiently gathering and monitoring data at an affordable rate. It is employed to look at and manage physical surroundings and transmit data to information systems. The methods and procedures of the proposed methodology have been laid out in Table 1.

**Table 1.**
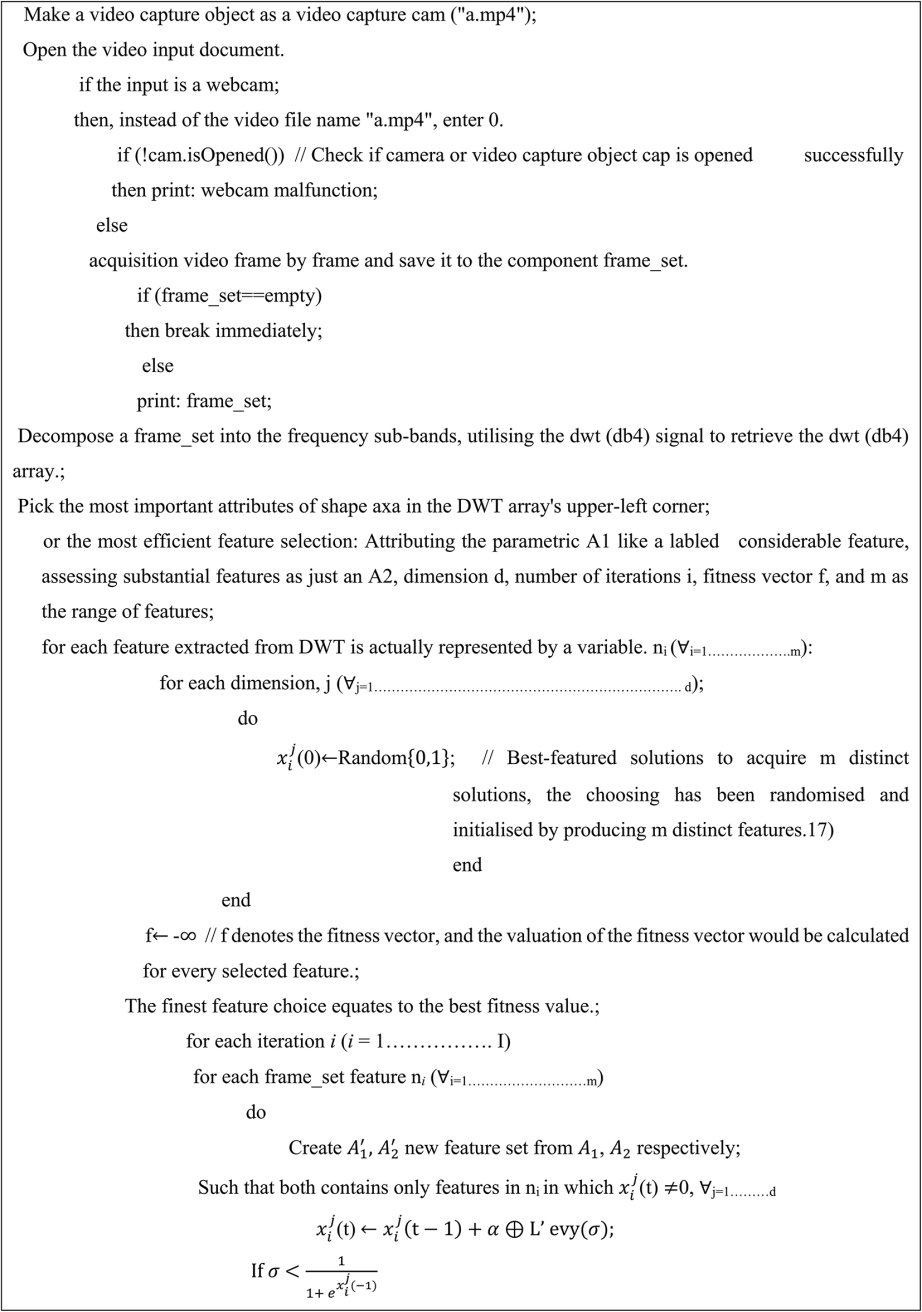

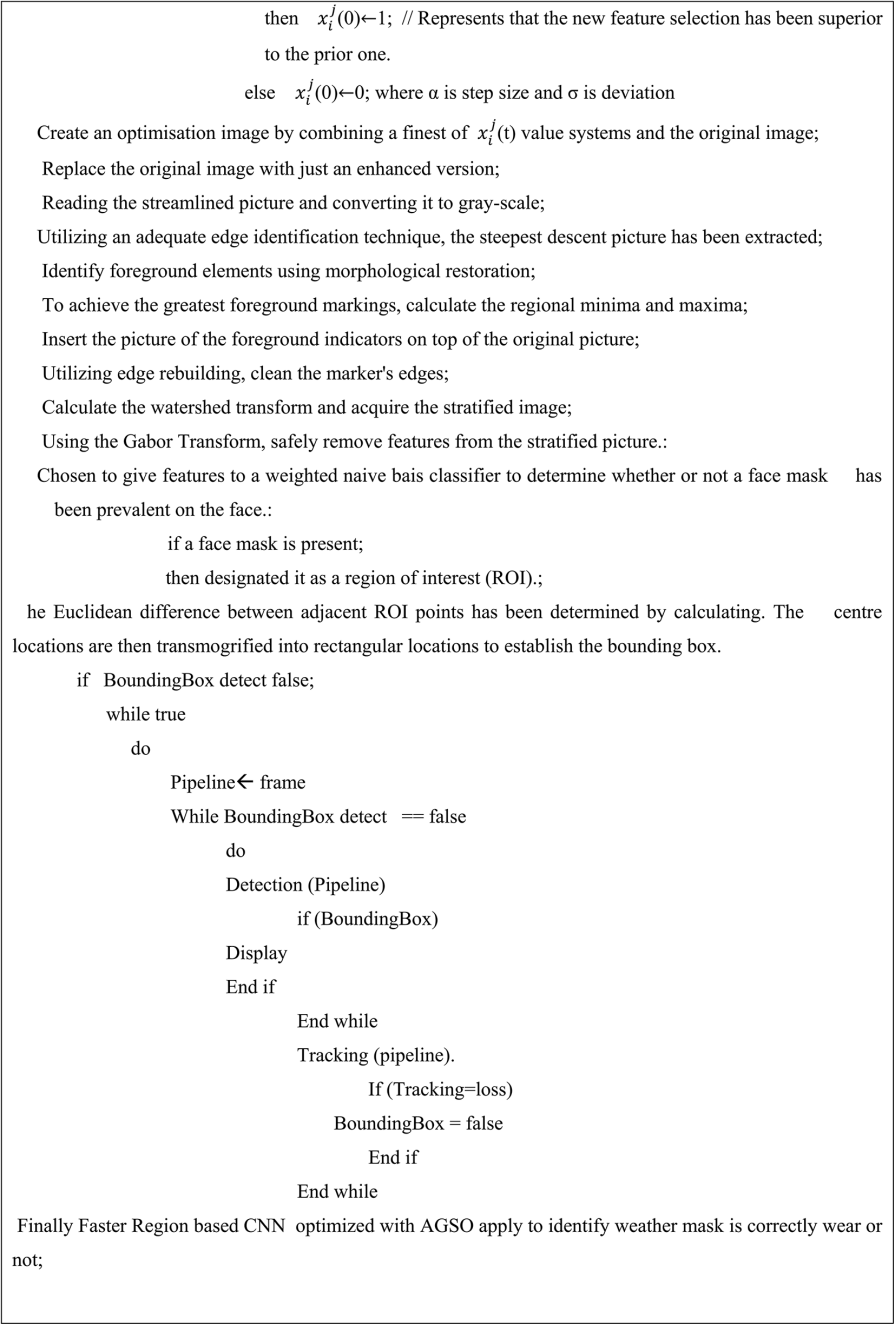
Step-by-step explanation of the methodology of proposed approach.

## Results and discussion

In order to make sure of the fact that the outcomes had only been pertinent to the identification model, every experiment of the proposed approach has been carried out on the MATLAB platform alongside TensorFlow and the PyTorch deep learning framework using an Intel(R) Core (TM) i7-8565U CPU @ 1.80 –1.99 GHz, RAM 8GB, Ubuntu 18.04 operating system. A variety of hyperparameters have been indicated for experimentation: dimensions = 20, population size = 20, iteration 1 = 300, iteration 2 = 300, learning rate = 0.5204, gradient = 0.1131, weight decay = 0.05131, and epoch number = 100, via the best training performance of 0.0978 at epoch number 81. Given an epoch number of 81, the proposed methodology provides the best training performance, with a mean square error of 0.097828. When the number of epochs exceeds 81, the run time increases while the mean square error does not decrease significantly. As a result, every experiment needed an epoch of 81. Furthermore, the mean square error of proposed approaches increases as the number of epochs exceeds 81. Throughout this section, multiple factors, including validation loss, validation accuracy, precision, recall, and F1-score, have been evaluated. A specific approach is being compared to other well-established methodologies applied to the same set of images under different circumstances. The work will present the final outcomes regarding the impact of wearing face masks, not wearing face masks, practicing social distancing, and the prevalence of individuals using authorised streaming video services.

### Dataset description

**Fig. 3:**
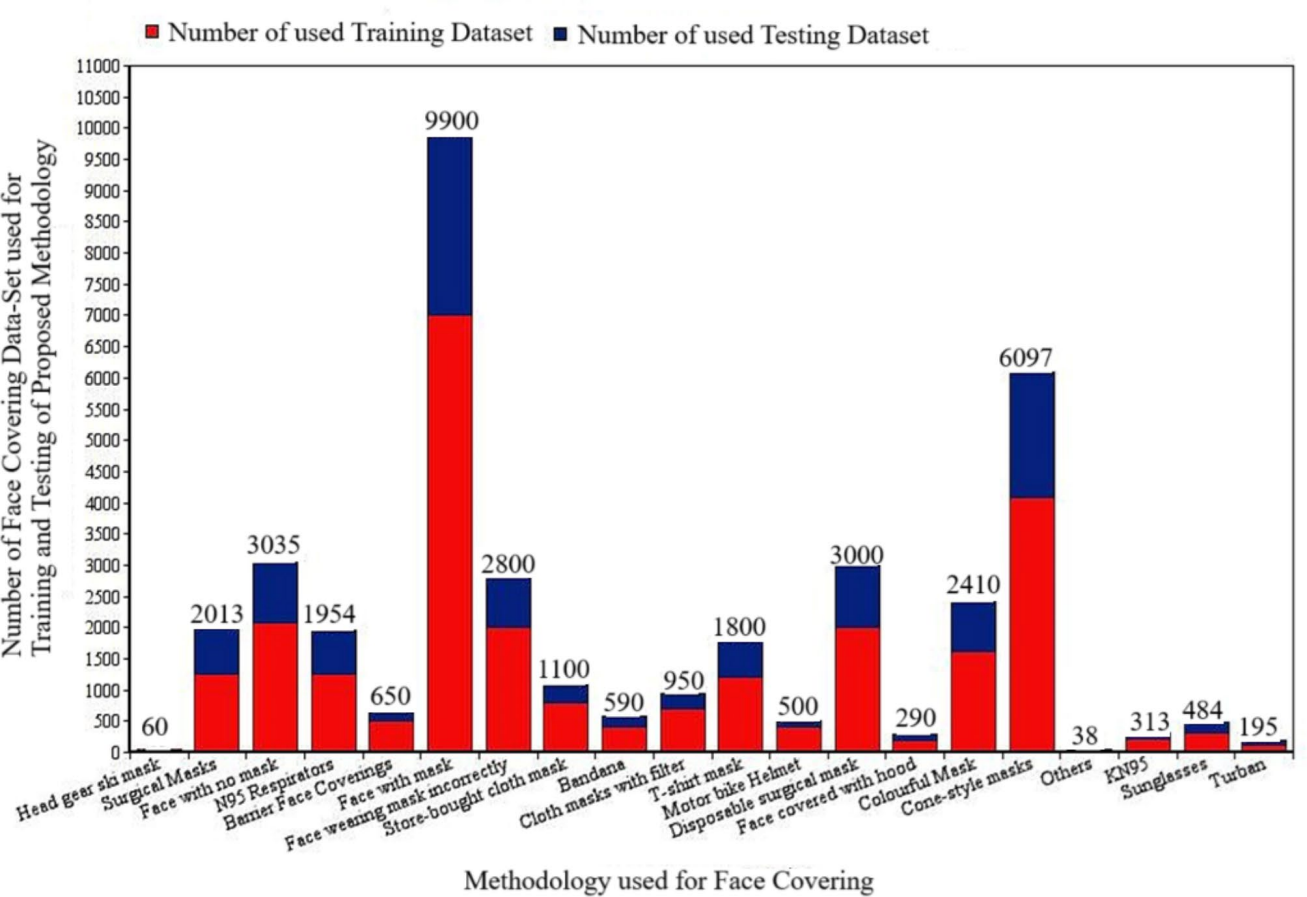
Number of training and testing dataset used for various methods of face covering.

**Fig. 4:**
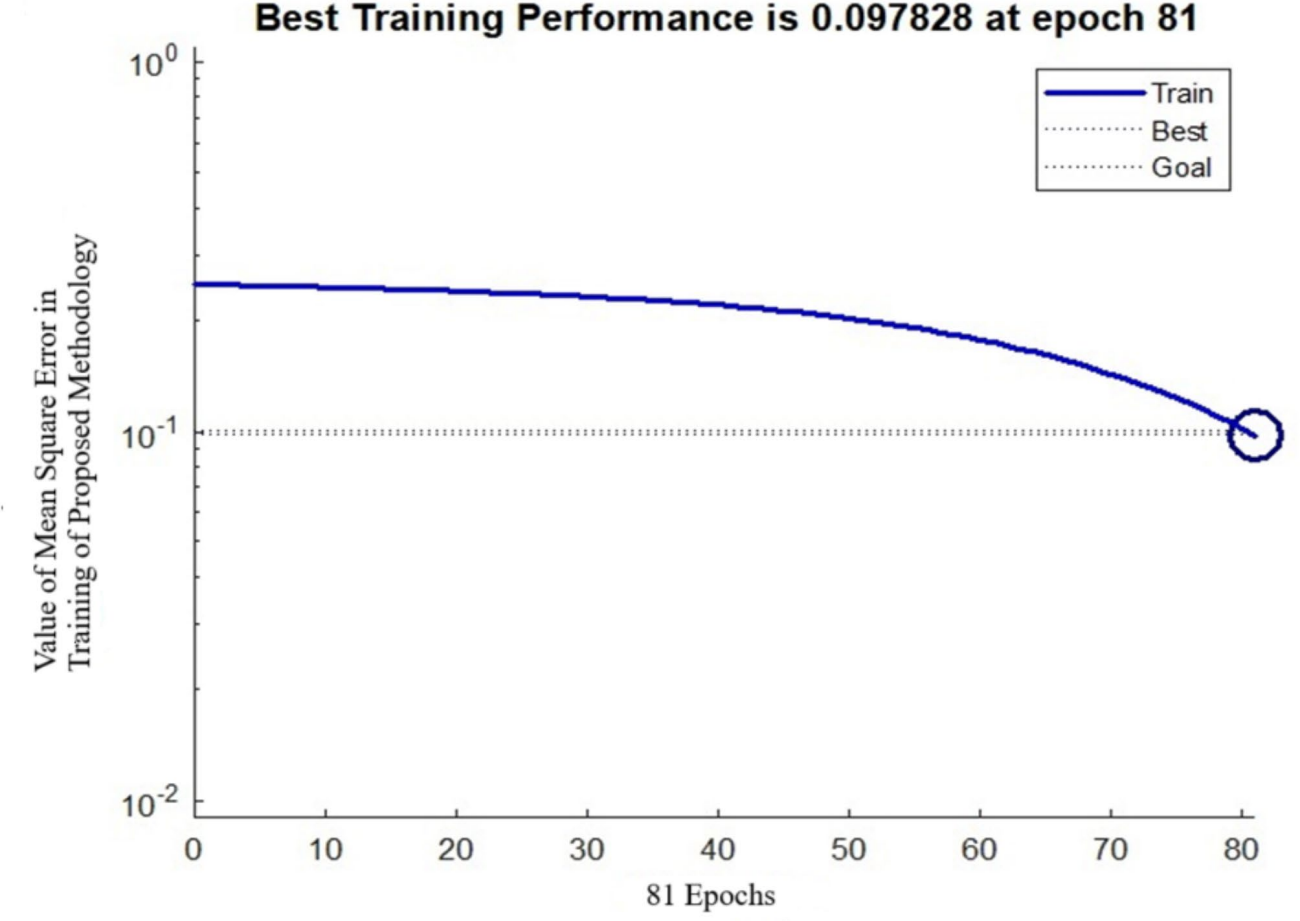
Details of best training performance.

**Fig. 5:**
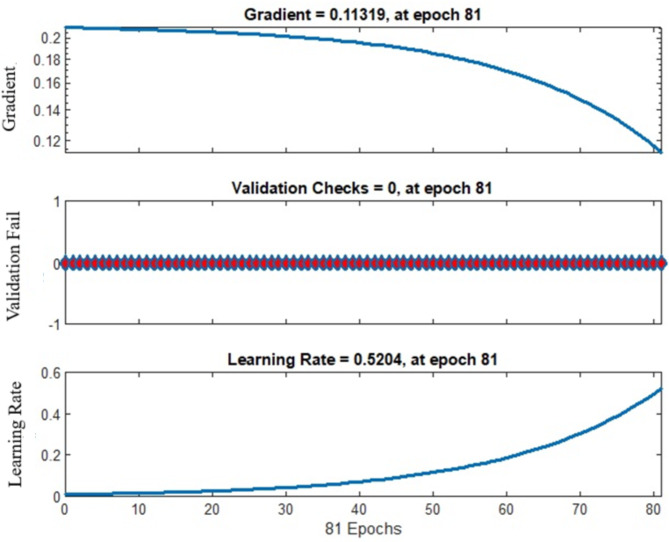
Details in the variation in accuracy of validation for training of proposed approach.

The proposed methodology utilizes a substantial amount of appropriate and incorrect face mask wear data sets collected through GitHub and Kaggle, which include the training repository of 16,000 images and a testing data set of 12,751 images. Each image has a variety of dimensions and backgrounds. For more effective recognition, images are captured in a variety of locations and lighting circumstances. To enhance the efficacy of the model’s training, the 10-fold cross-validation method is going to be used. The standard image data set illustrating appropriate and incorrect face mask wear has been thoroughly divided into ten separate groups using the ten-fold technique. The remaining subgroup is included in the test data, while the set of training data for every iteration of the models is made up of nine subgroups. To reduce bias, a substantial amount of the available data set is utilized to train the models over ten iterations. Furthermore, the model weights of the convolutional layers are constantly altered during every iteration, thus increasing the learning procedure’s efficiency. When the total amount of visuals is low, model accuracy suffers, along with the risk of overfitting. This suggested methodology employs data augmentation techniques to expand the dataset’s size in order to prevent these issues. Data augmentation techniques have significance for datasets with few images because they improve not only the size of the dataset but also the variety of input images, causing the model that was created able to adapt to complicated backgrounds and enhancing its robustness. Color change and geometric transformation techniques are frequently employed in data augmentation. Color transformation approaches include contrast, hue, and saturation transformation, while geometric transformation methods consist of random scaling, flipping, and cropping. Table 2 displays a range of techniques for face covering in the dataset, along with the amount of data used for training and testing. Figure 3 exhibits diverse categories within a test dataset. Figure 4 depicts the model’s training performance at its best, while Fig. 5 showcases the variability of validation accuracy for different training sets.

**Table 2:** Training and test data sets based on face wrapping techniques.

S. No.	Methodology used for face covering	Number training dataset of used for each face covering methodology	Number testing dataset of used for each face covering methodology
1	Head gear ski mask	40	20
2	Surgical Masks	1250	763
3	Face with no mask	2081	954
4	N95 Respirators	1254	700
5	Barrier Face Coverings	500	150
6	Face wearing mask	7000	2900
7	Face wearing mask incorrectly	2000	800
8	Store-bought cloth mask	800	300
9	Bandana	400	190
10	Cloth masks with filter	700	250
11	T-shirt mask	1200	600
12	Motor bike Helmet	400	100
13	Disposable surgical mask	2000	1000
14	Face covered with hood	200	90
15	Colourful Mask	1618	792
16	Cone-style masks	4093	2004
17	others	22	16
18	KN95	210	103
19	Sunglasses	325	159
20	Turban	131	64

### Validation loss

The training loss is used to measure the inaccuracies of the training dataset mentioned earlier, and it is evaluated at each epoch. The validation inaccuracy primarily stems from the transfer of the validation dataset during the training process, specifically assessed after each epoch. An epoch is a term used to describe a series of operations or iterations carried out on the complete training dataset. Equation (1) can be used to compute the validation loss using the training loss.

3 $$\:\text{V}\text{a}\text{l}\text{i}\text{d}\text{a}\text{t}\text{i}\text{o}\text{n}\:\text{L}\text{o}\text{s}\text{s}=\:\frac{\text{T}\text{r}\text{a}\text{i}\text{n}\text{i}\text{n}\text{g}\:\text{L}\text{o}\text{s}\text{s}}{\text{T}\text{r}\text{a}\text{i}\text{n}\text{i}\text{n}\text{g}\:\text{I}\text{m}\text{a}\text{g}\text{e}\text{s}\:}\:\times\:1000$$

**Fig. 6:**
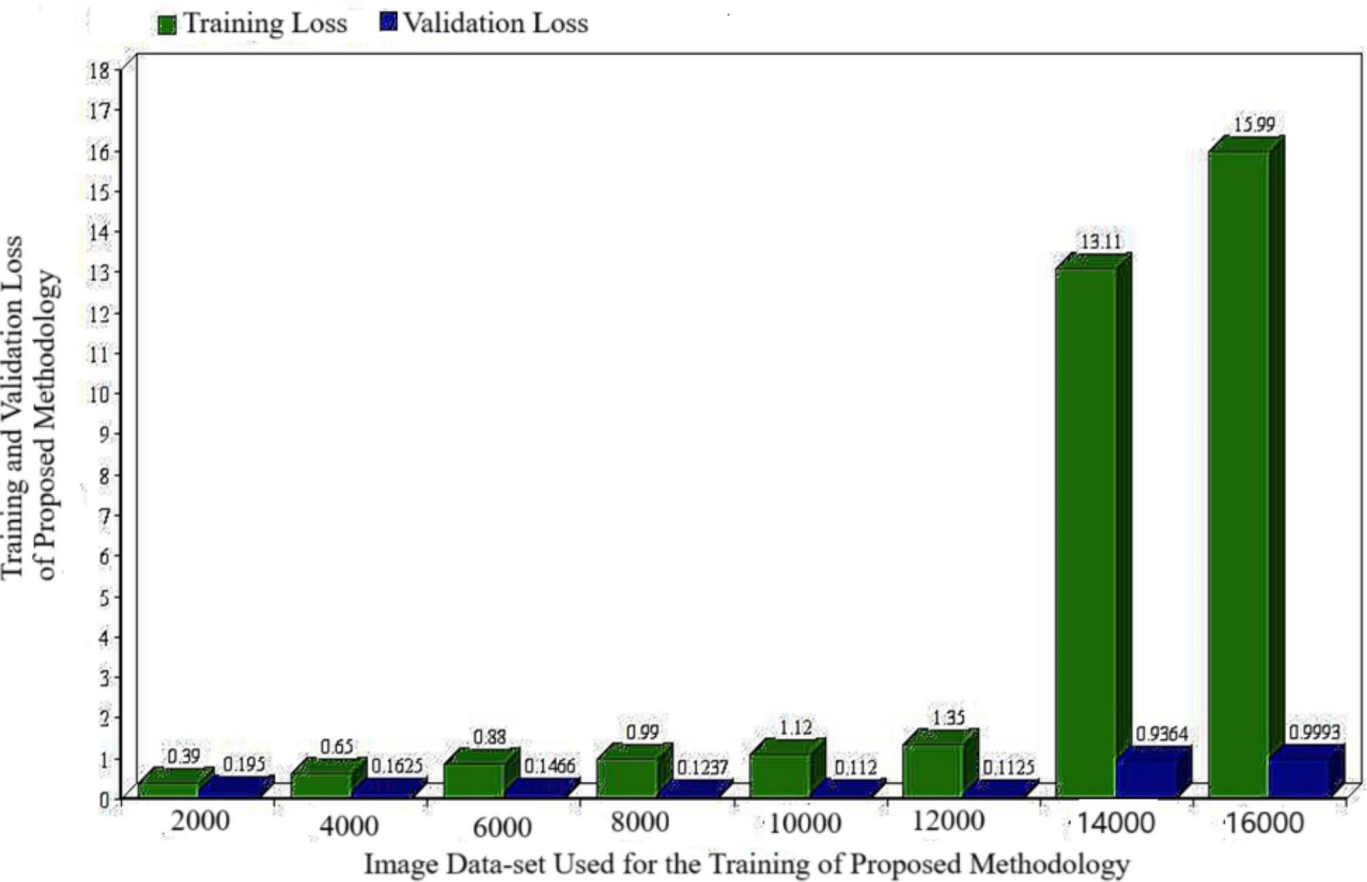
Training and validation loss variability of proposed methodology.

The loss in training and validation are evaluated to some extent based on the mean square error. Table 3 presents the training and validation loss for a multitude of training images. It is evident that the validation error remains constant or increases while the training error decreases from 12,000 to 2000 images. Indeed, the term “overfitting” is used to describe such a circumstance. Everything is in order after the completion of the 12,000-test set, as both are progressing along the same trend. Furthermore, Fig. 6 demonstrates the relationship between the validation loss and the number of training samples. The validation loss initially decreases as the number of training data images increases, but it significantly increases once it reaches 14,000.

**Table 3:** Training loss and validation loss of proposed methodology.

S. No.	Images Data-set used in Proposed Methodology	Training Loss of Proposed Methodology	Validation loss of Proposed Methodology
1	2000	0.39	0.1950
2	4000	0.65	0.1625
3	6000	0.88	0.1466
4	8000	0.99	0.1237
5	10,000	1.12	0.1120
6	12,000	1.35	0.1125
7	14,000	13.11	0.9364
8	16,000	15.99	0.9993

### Accuracy in validation

**Fig. 7:**
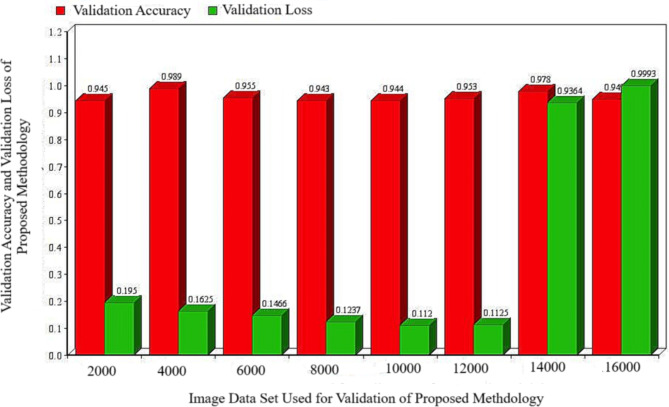
Comparison of validation loss and validation accuracy of proposed methodology.

The accuracy of validation is a crucial metric for forecasting algorithm effectiveness. A model has been validated by assessing both the loss and consistency metrics. Table 5 displays, a comparison of Validation loss and Validation Accuracy of Proposed Methodology. The validation accuracy, as indicated in the last column, has consistently been below 1. This value is expressed as a percentage by multiplying the given evaluation by 100. The variability of validation accuracy persists, despite the reduction of the validation deficit through the augmentation of training examples from 4000 to 14,000. Consequently, the aforementioned paragraph has acquired the label of “overfitting.” In addition, as the quantity of pictures surpasses 16,000, the level of accuracy gradually diminishes. It indicates that the framework has been thoroughly mastered. Figure 7 illustrates the relationship between the accuracy of validation and the increasing number of training data images.

**Table 4:** Comparison of validation loss and validation accuracy of proposed methodology.

S. No.	Images data-set used in proposed methodology	Validation loss of proposed methodology	Validation accuracy of proposed methodology
1	2000	0.1950	0.945
2	4000	0.1625	0.989
3	6000	0.1466	0.955
4	8000	0.1237	0.943
5	10,000	0.1120	0.944
6	12,000	0.1125	0.953
7	14,000	0.9364	0.978
8	16,000	0.9993	0.949

### Performances metrices

The suggested methodology utilises True Positives (TP), False Positives (FP), True Negatives (TN), and False Negatives (FN) to compute performance metrics such as precision and recall^52^. Precision is the quotient of accurately predicted favourable experimental data divided by the total number of accurately predicted positive observations, while recall is the proportion of accurately predicted positive observational data to the total number of samples. The F1-score was computed by taking the harmonic mean of the recall and precision.

4 $$\text{Precision}=\:\frac{\text{T}\text{P}}{\text{T}\text{P}+\text{F}\text{P}}$$

5 $$\text{Recall}=\:\frac{\text{T}\text{P}}{\text{T}\text{P}+\text{F}\text{N}}$$

.

**Fig. 8:**
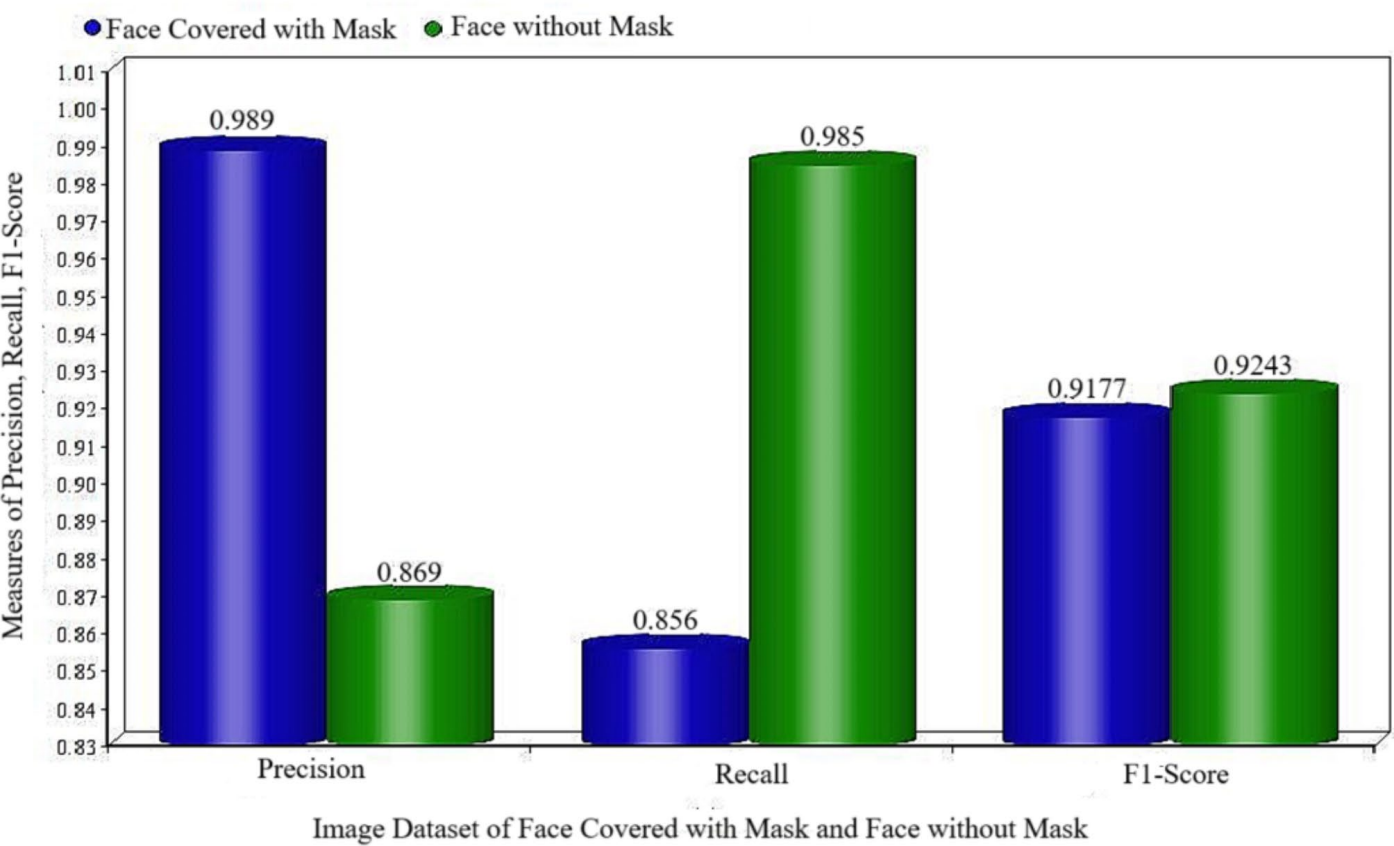
Precision, recall, and F1-score evaluation on testing image data set of face covered with mask and face without mask.

Table 5 illustrates the precision, recall, and F1-score for a specific test set. The dataset labelled “With Mask” includes the data sets numbered 1, 6, 15, and 16 in Table 2. The dataset labelled “Without Mask” includes all the remaining data sets. Figure 8 provides a visual representation of the comparison between precision, recall, and F1-score for testing datasets with and without wearing masks. The new methodology demonstrates excellent precision, recall, and F1-score for both types of datasets.

**Table 5:** Precision, recall, and F1-score for the testing dataset.

Dataset	Precision	Recall	F1-Score
Face Cover with Mask	0.989	0.856	0.9177
Face Without Mask	0.869	0.985	0.9243

### Evaluation of the suggested methodological approach to the available method

**Fig. 9:**
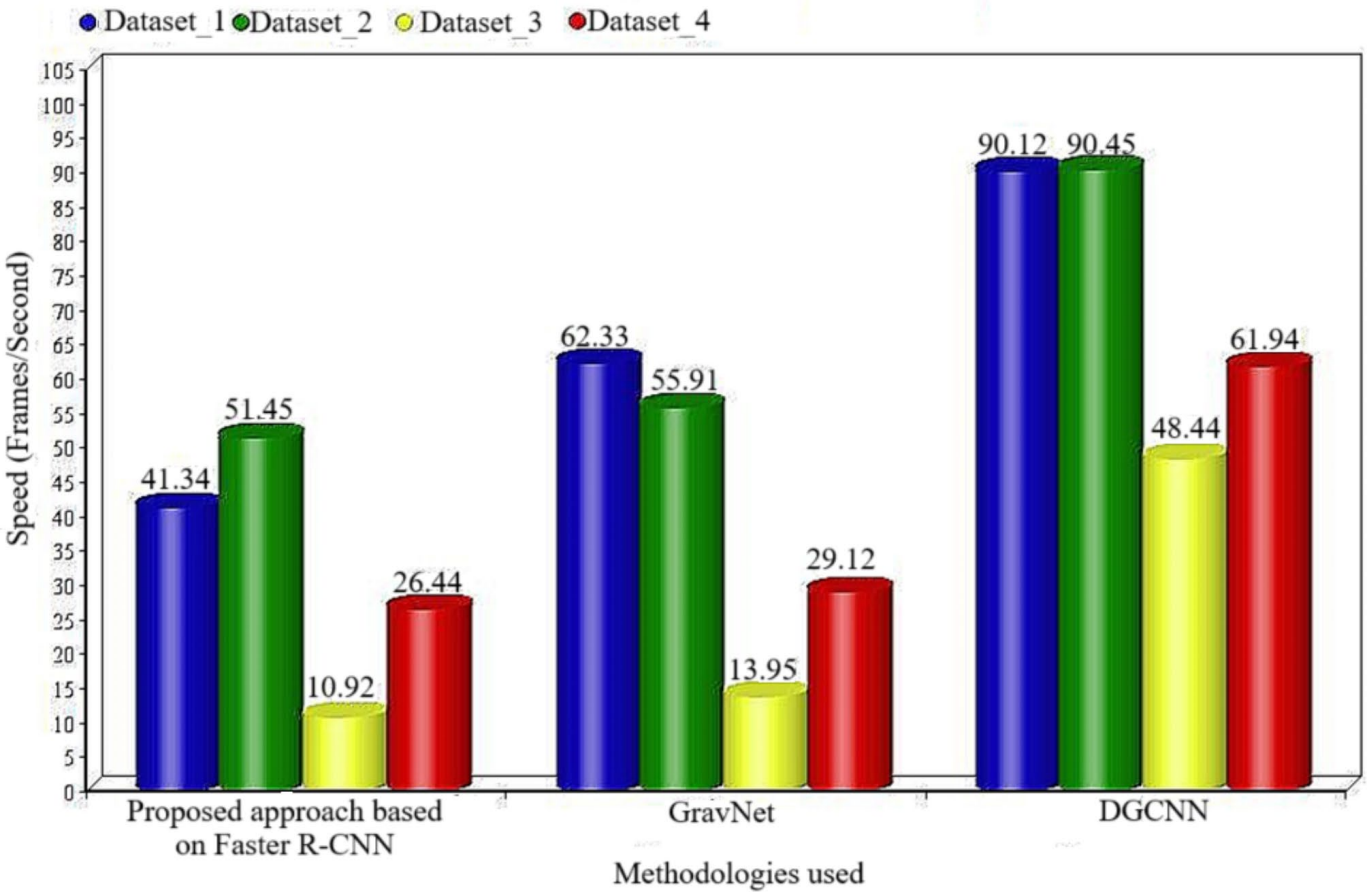
Comparison (in terms of speed in fps) of Faster R-CNN with other Techniques.

The proposed approach based on Faster R-CNN, has demonstrated superior performance when compared to other methods applied to the same dataset. Table 6presents a comparative analysis of speed, measured in frames per second. The data collection process will be divided into four datasets, namely Dataset 1, Dataset 2, Dataset 3, and Dataset 4 as per Fig. 9 . These datasets correspond to a college campus, a traffic area, a street, and a shopping mall, respectively. Studies have demonstrated that Faster R-CNN outperforms other techniques, including GravNet and DGCNN approaches^53^. Figure 8 illustrates a comparative assessment of the speed of concepts in frames per second (fps) between Faster R-CNN and other methods.

**Table 6:** A comparison of faster R-CNN to other technologies in terms of speed in frames per second.

Technique	Dataset_1	Dataset_2	Dataset_3	Dataset_4
Proposed approach based on Faster R-CNN	41.34	51.45	10.92	26.44
Single Shot Detector	59.44	54.25	12.15	27.88
GravNet	62.33	55.91	13.95	29.12
DGCNN	90.12	90.45	48.44	61.94

### Outcomes for face-masking, social distancing, and counting in real time

**Fig. 10:**
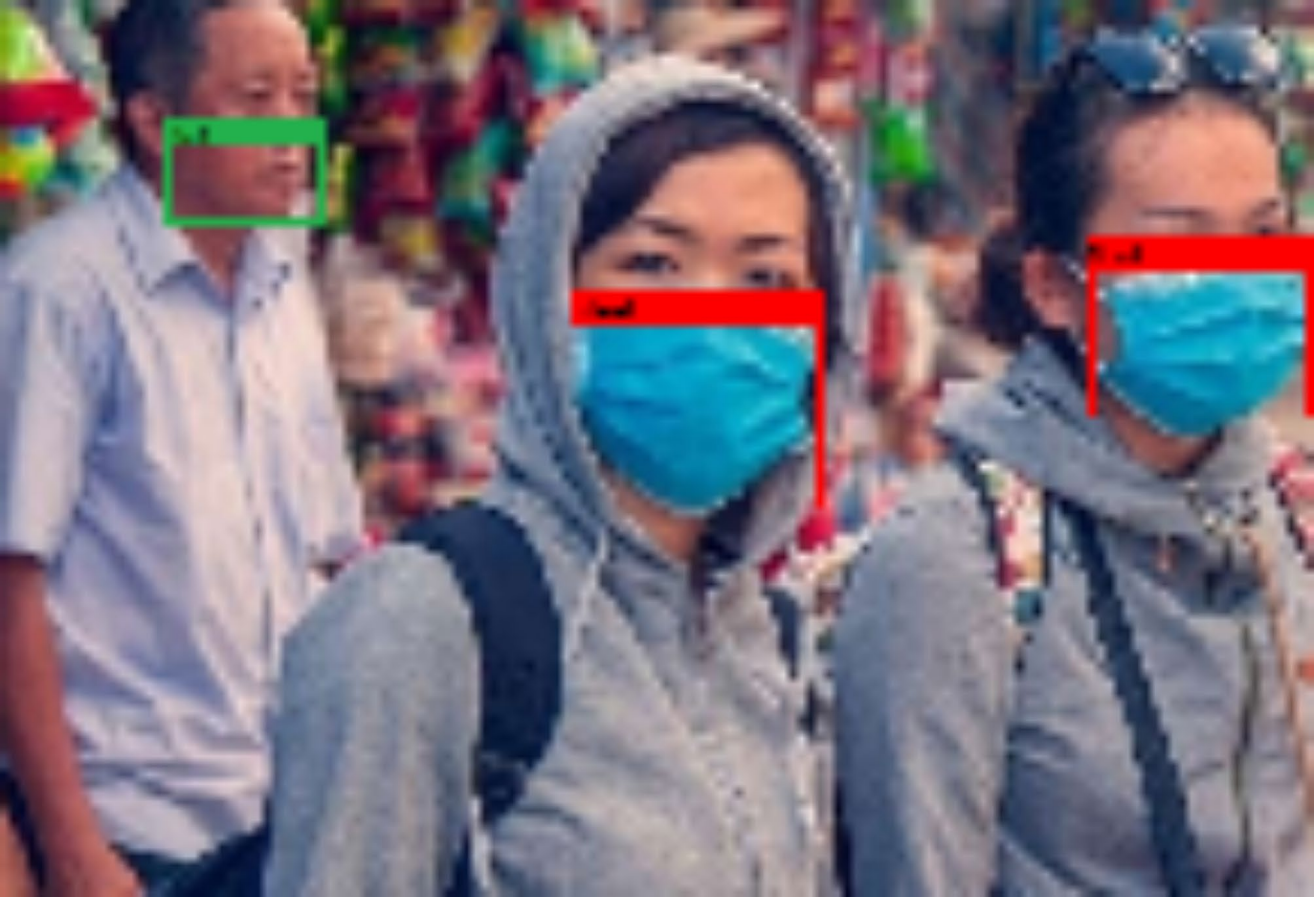
Mask count is represented by pink, and the unmask count is shown in green.

**Fig. 11:**
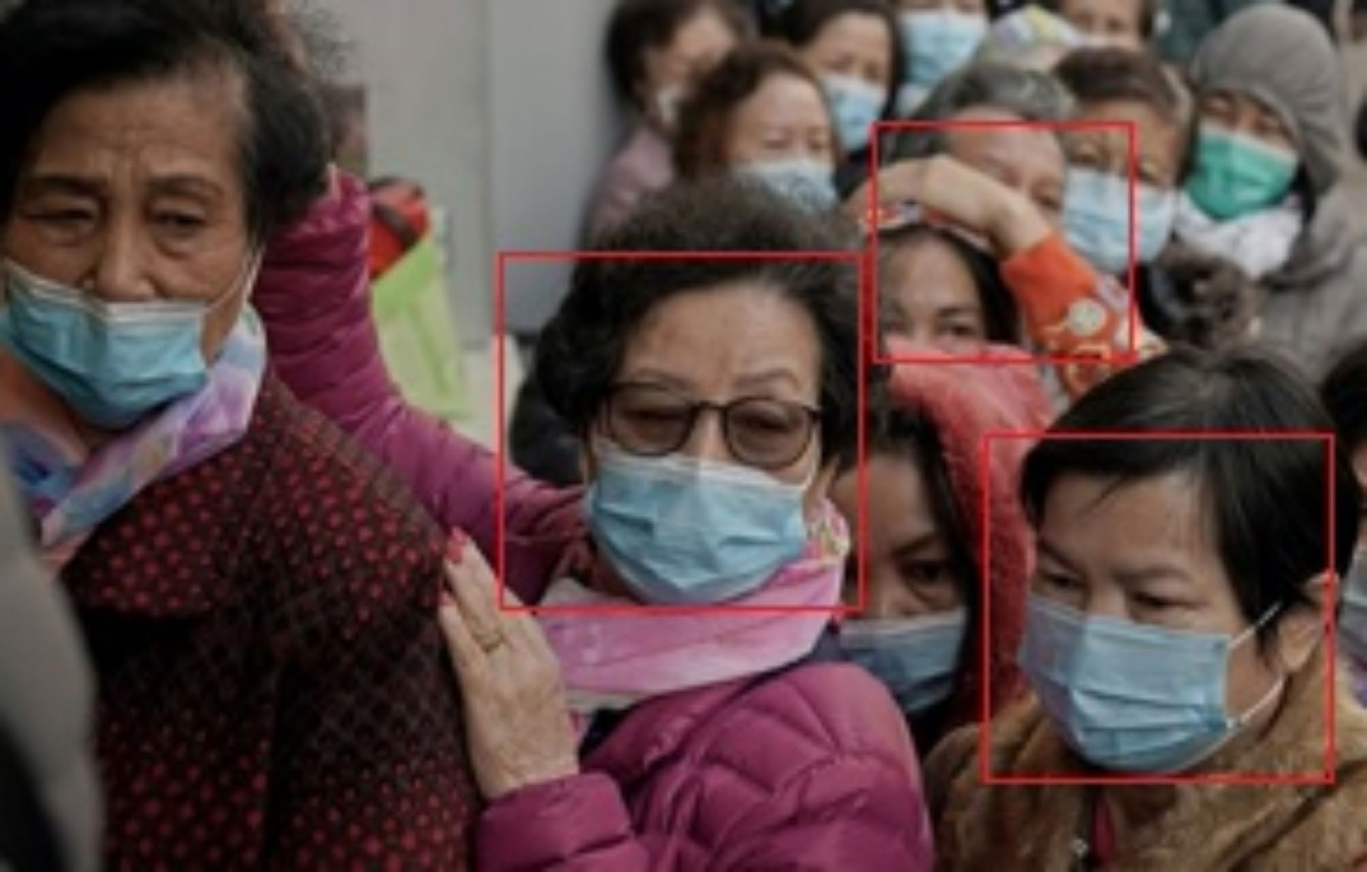
Maintaining social distance and detecting of red objects.

The majority of UAVs are equipped with artificial intelligence and deep learning algorithms, which enhance their ability to perform tasks and increase their accuracy. Advanced unmanned aerial vehicles (UAVs) are able to obtain aerial imagery within real-time constraints. The utilisation of UAVs can greatly decrease the duration of real-time computing processes. The utilisation of libraries such as OpenCV, PyTorch, Tensor Flow, and Keras proved indispensable in effectively resolving live video reliability concerns and providing assistance in addressing object recognition challenges. Figure 10illustrates the instantaneous representation of the mask and unmask numbers. The UAV-captured image would undergo a conversion to grayscale, followed by an enhancement of its sharpness. Image contrast in the initial phase has been attained through the utilisation of Optimal Wavelet-Based Masking (OWBM) and the Enhanced Cuckoo Search Algorithm (ECSA). The distinguishing characteristics for differentiating individuals who wear masks from those who do not wear masks have been extracted from enhanced image frames in the second stage, employing Gabor transform and stroke width transform algorithms. In the third stage, a weighted naive Bayes classifier (WNBC) was utilised to accurately identify the presence of a face mask within a scene image based on the derived characteristics. The fourth stage utilises the faster R-CNN^54^technique, which is a deep neural network-based convolutional neural network. It employ AGSO to identify and rectify instances of incorrect mask donning in UAV images. Similarly, the process of measuring distance took place by employing detection and tracking technologies^55^, where each individual can be recognised in real-time using bounding box coordinates. Figure 10 illustrates the mask-wearing status, with green indicating the absence of a mask and red indicating proper mask usage. Figure 11 illustrates the process of determining social distance in a Kaggle dataset. The presence of red artefacts indicates that these artefacts have a relatively short distance and are not safe to approach.

## Technical challenges faced during implementation

The main challenge faced during implementation is the dataset of images used in this proposed method, which exhibits limited variation within each class and low pixel density. Optimising the weights to achieve the most effective solutions and creating a highly functional and efficient Convolutional Neural Network (CNN) with minimal or maximal impact on the variables involved in the error function is a laborious task due to the complex landscape of ups and downs. There is also a technological challenge in the implementation process that needs to be solved by doing more research to get better frame data sets using OWBM and the Enhanced Cuckoo Search Algorithm (ECSA).

## Conclusion

The whole world has undeniably experienced significant repercussions due to the outbreak of the coronavirus disease in 2019. It is advisable for individuals to wear a mask in public spaces as a substantial measure of protection. In order to impede the transmission of the virus, numerous regions implemented a compulsory policy requiring individuals to wear masks in densely populated areas. Multiple researchers have conducted investigations on image-based automated facial mask identification. This work involves the development of a sophisticated Unmanned Aerial Vehicle (UAV) that utilises advanced deep learning techniques and an improved version of the cuckoo optimisation algorithm. The primary objective of this UAV is to detect masks and track social distancing. Without an Unmanned Aerial Vehicle (UAV), it becomes more challenging to differentiate between a group of individuals who are genuinely not wearing masks and those who are not adhering to social distancing measures. A UAV, or unmanned aerial vehicle, was employed in this research to detect various objects using the faster R-CNN algorithm as well as YOLOv3. The researchers have presented a comprehensive perspective on the identification of individual face masks and the monitoring of violations of social distancing. The enforcement of face-mask usage and adherence to social distancing guidelines are two crucial measures for mitigating the transmission of COVID-19. The proposed research consists of three main components. Firstly, it involves classifying face masks using a weighted naive Bayes classifier (WNBC) and a deep learning-based, faster region-based convolutional neural network (Faster R-CNN) optimized AGSO to identify appropriate and incorrect face-mask wear in UAV images. Secondly, it focuses on tracking social distancing through object recognition and traceability strategies. This involves real-time recognition of individuals using bounding boxes. During our face mask recognition comparison, the faster R-CNN model outperformed the Single Shot Detector, GravNet, and DGCNN models in terms of speed and accuracy.

The results showed that the face cover with mask achieved a training precision of 98.9%, a recall of 85.6%, and an F1 score of 91%. On the other hand, the Face Without Mask achieved a training precision of 86.9%, a recall of 98.4%, and an F1 score of 92.43%. It is important to highlight that the validation error for the entire collection of 16000 images remains the same or increases, while the training error increases from 12000 to 2000 images. The term for this problem appears to be overfitting. After 12000 training examples, the alignment between the two entities appears to be flawless as they are both following a congruent trajectory. The validation loss initially decreases to a low level as the number of training photos increases, but once more than 14,000 images are used for training, the validation error starts to increase rapidly. Consequently, the effects of social distancing were evaluated on different sets of videos, with crowded areas specifically taken into account to enhance the difficulty of recognition. Moreover, the article presents an expedited R-CNN methodology and demonstrates that object recognition is significantly faster compared to other established, sophisticated techniques. The validation loss, validation accuracy, precision, and recall of the proposed approach have been determined to be optimal.

.

## Data Availability

The datasets used and/or analysed during the current study available from the corresponding author on reasonable request.
